# Randomized controlled open-label trial to evaluate prioritization software for the secondary triage of patients in the pediatric emergency department

**DOI:** 10.1186/s12245-024-00623-3

**Published:** 2024-04-08

**Authors:** Thomas Lun, Jessica Schiro, Emeline Cailliau, Julien Tchokokam, Melany Liber, Claire de Jorna, Alain Martinot, François Dubos

**Affiliations:** 1 Pediatric Emergency Unit & Infectious Diseases, Univ. Lille, CHU Lille, Lille, F-59000 France; 2 INSERM, CIC-IT 1403, Lille, F-59000 France; 3 Department of Biostatistics, CHU Lille, Lille, F-59000 France; 4 Univ. Lille, METRICS: Évaluation des technologies de santé et des pratiques médicales - ULR 2694, Lille, F-59000 France

**Keywords:** Patient flow, Patient prioritization, Pediatric emergency department, Second triage, Usage assessment

## Abstract

**Background:**

The continual increase in patient attendance at the emergency department (ED) is a worldwide health issue. The aim of this study was to determine whether the use of a secondary prioritization software reduces the patients’ median length of stay (LOS) in the pediatric ED.

**Methods:**

A randomized, controlled, open-label trial was conducted over a 30-day period between March 15th and April 23rd 2021 at Lille University Hospital. Work days were randomized to use the patient prioritization software or the pediatric ED’s standard dashboard. All time intervals between admission and discharge were recorded prospectively by a physician not involved in patient care during the study period. The study’s primary endpoint was the LOS in the pediatric ED, which was expected to be 15 min shorter in the intervention group than in the control group. The secondary endpoints were specific time intervals during the stay in the pediatric ED and levels of staff satisfaction.

**Results:**

1599 patients were included: 798 in the intervention group and 801 in the control group. The median [interquartile range] LOS was 172 min [113–255] in the intervention group and 167 min [108–254) in the control group (*p* = 0.46). In the intervention group, the time interval between admission to the first medical evaluation for high-priority patients and the time interval between the senior physician’s final evaluation and patient discharge were shorter (*p* < 0.01). The median satisfaction score was 68 [55–80] (average).

**Conclusion:**

The patients’ total LOS was not significantly shorter on days of intervention. However, use of the electronic patient prioritization tool was associated with significant decreases in some important time intervals during care in the pediatric ED.

**ClinicalTrials.gov**: NCT05994196

**Trial registration number**: NCT05994196. **Date of registration**: August 16th, 2023

**Supplementary Information:**

The online version contains supplementary material available at 10.1186/s12245-024-00623-3.

## Introduction

The continual increase in patient attendance at the emergency department (ED) is a worldwide health issue [1–3]. Overcrowding in the ED leads to an increase in a patient’s length of stay (LOS), induces patient dissatisfaction, creates stress for the ED staff, and increases the risk of medical errors [4–6]. A number of patient flow strategies have been developed [4], including walk-in centers close to the ED, triage by nurses or physicians, nurse flow coordinators, a fast-track area, and bed managers. It has been suggested that the implementation of a triage liaison physician can limit bottlenecks at certain stages of patient care and thus reduce overcrowding and increase throughput [7]. In some adult EDs, a LOS of less than 4 h is a quality target; this is the case in Australia, for example, with the National Emergency Access Target [8]. After the initial triage and once patient management has started, secondary prioritization is often disorganized; a patient may sometimes wait for a long time before the next step in their diagnosis or treatment.

A software package called Optimum® was developed in the pediatric ED (PED) at Lille University Medical Center (Lille, France). After initial triage by a nurse, Optimum® serves as a secondary triage for physicians and other staff by optimizing their subsequent actions [9].

The primary objective of the present study was to assess the effect of using Optimum® on the patients’ median LOS in the PED. The secondary objectives were to determine Optimum®’s effects on (i) the number of patients present in the PED simultaneously, (ii) the respective time intervals between PED admission and the first medical contact (with a medical student), the first evaluation by a senior physician, the first evaluation by a surgeon or pediatric subspecialist, the provision of radiological or blood test results, and discharge, and (iii) the PED staff’s level of satisfaction.

## Methods

### Design and inclusion criteria

A randomized, controlled open-label trial was conducted in the PED at Lille University Hospital between March 15th and April 23rd, 2021 (NCT05994196), following CONSORT guidelines (Supplementary Material 1). Each day was randomized for the use of Optimum® (i.e., the interventional group) vs. the PED’s standard patient management dashboard (the control group). We assumed that randomization would distribute equally between the two groups patients who were or were not time-consuming for the medical staff. As only 23% of visits took place between midnight and 10 am, with no impact on patient flow, children admitted between 10 am and midnight were included in the analysis. Patients who left without being seen by medical staff and those subsequently admitted to a short-stay unit were excluded. The study protocol was registered with the French National Data Protection Commission (*Commission Nationale de l’Informatique et des Libertés*, Paris, France; registration number: DEC21-056). In line with the French legislation on analyses of anonymized data from clinical practice, approval by an institutional review board was not required. The patients and their parents were shown a study information sheet at the PED reception desk and were free to object to their child’s participation.

### Setting and definitions

The trial was carried out in our tertiary care center, which receives almost 30,000 PED visits annually, mainly Caucasians. One third of children (0–16 years of age) had an underlying condition and 1/3 needed orthopedic or surgical management. The mean waiting time before being seen by a physician was 1h36 and the mean LOS was 3h25 in 2022 for those without further admission. From 2019 to 2021, 0.9 to 1.6% of patients left the ED without being seen. Physicians working in the PED were pediatricians and the triage at admission was performed by a nurse.

The primary goal of triage is to ensure that patients receive timely, appropriate care, maximizing positive outcomes and minimizing potential harm. Primary triage is a process to determine the urgency of further care at the time of patient arrival [10]. Several triage systems are used, including the Canadian triage scale in our PED. Secondary triage is a new concept for the ED, designed to enable patients to be organized, monitored and assessed effectively [11], even after the initial triage. The concept is developed in this trial with a new tool.

### Standard dashboard and secondary triage tool

The standard patient management dashboard provides a list of patients in order of arrival with, next to the name, the length of time the patient has been on the unit, the level of urgency (color-coded triage), any tests or medical advices prescribed to be carried out or completed, and the initials of the staff in charge of the patient.

**Fig. 1:**
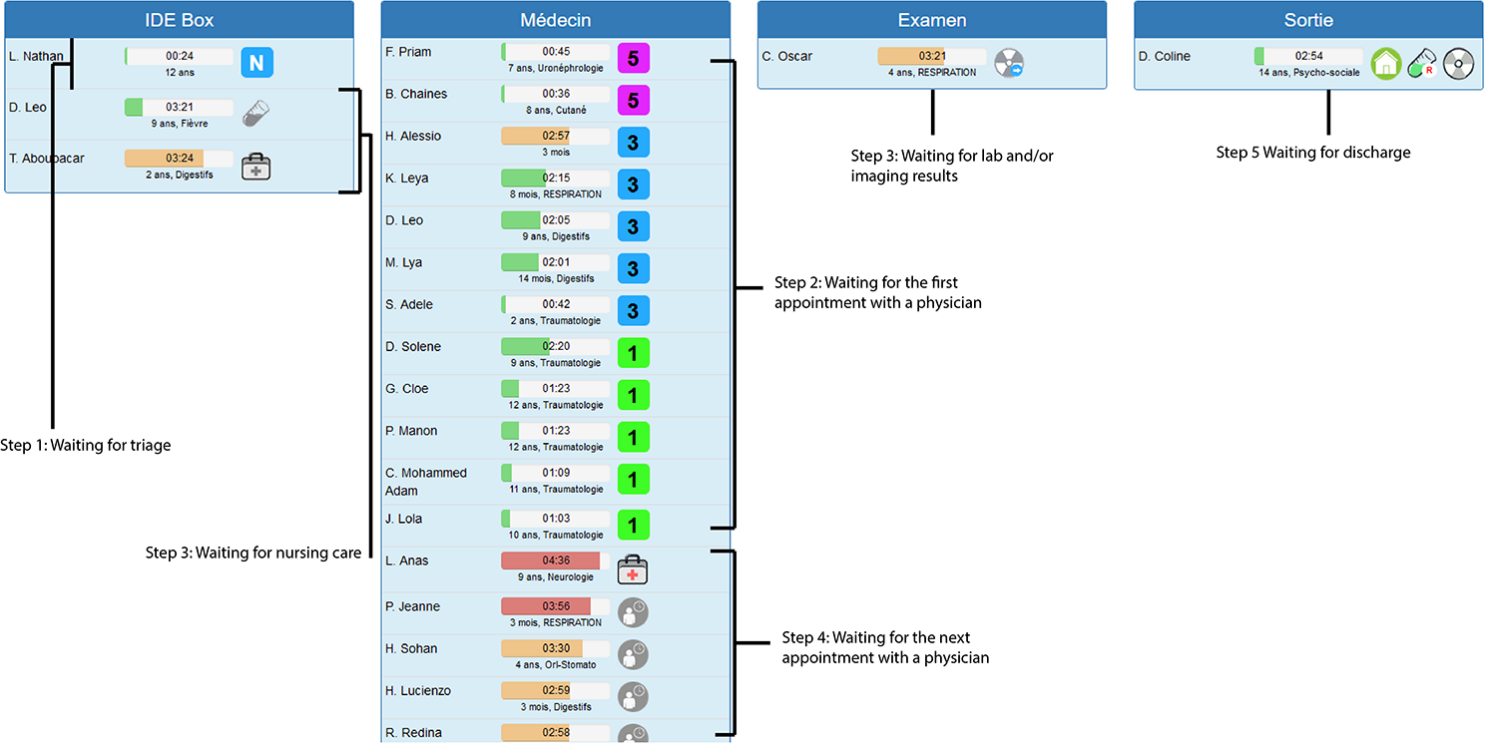
The Optimum software’s interface and annotations on its use. The colored status bar next to the patient’s name corresponds to the LOS in the PED. The status bar’s color depends on the patient’s LOS, relative to that of patients admitted for the same reasons. The bar is green when the patient’s LOS is below the 50th percentile but changes to yellow when the LOS is between the 50th and 75th percentiles, to red when the LOS is between the 75th and 95th percentiles, and lastly to dark red (overcrowding) when LOS is greater than the 95th percentile. Once the PED is overcrowded, the priority is discharging patients rather than seeing new ones

Optimum® was a proof-of-concept software developed in 2015 [12]. Starting from a database of 75,000 visits, 100 different reasons for PED visits were retrospectively defined, and the LOS in the PED was determined for each. The five variables with a statistically significant influence on the LOS were the reason for PED visit, the number of patients present in the ED simultaneously, the prescription of imaging, the prescription of blood tests, and the prescription of treatment [13]. The Optimum®’s interface is shown in Fig. 1. The data in the PED’s standard patient management dashboard required by Optimum® were transferred to Optimum® within five minutes in 83% of cases and within 10 min in 94% of cases [12].

Optimum®’s purpose is to remove the mental load of prioritization from the PED staff. The software first prioritizes the triage of new patients by the ED staff and the first evaluation of a new patient by the medical team. Optimum® then prioritizes blood sample collections and care (for nurses) and review of imaging results, blood test results or an evaluation by a specialist (for physicians). Lastly, Optimum® prioritizes the final step in patient management by a senior physician, when appropriate. It means that a character on a grey background (see Fig. 1, column 2, last thumbnail) appears when all the other actions have been completed, meaning that a decision by the senior physician is awaited: either a blood test, or a new treatment, or to validate a discharge.

### Endpoints and the number of patients needed

The study’s primary endpoint was the LOS for each patient. We hypothesized that there would be a 15-minute difference in the LOS between the intervention and control groups. This would correspond to one less patient in the PED at a given time, when considering 28,500 PED visits per year (78 per day) and a median LOS of 190 min. To assess a median 15-minute difference in LOS between the two groups with an α-risk of 0.05 and a power (1-ß risk) of 0.8, we calculated that a total of 1542 patients had to be included (i.e., 771 per group). The secondary endpoints were the number of patients present at the same time in the PED per day and per period of the day, the time intervals between each stage in patient management from nurse triage to discharge, and the PED staff’s level of satisfaction.

### Study procedures and data collected

During the two weeks prior to the start of the study, all the PED staff members were trained in use of Optimum®. This secondary triage tool was set up in the PED at this time, so that all the staff members could familiarize themselves with the tool and put any questions to the investigators.

The days were randomized to Optimum® vs. the standard dashboard using the “random” formula in Excel® (Microsoft Corporation, Redmond, WA, USA). Firstly, the study dates were entered into an Excel® spreadsheet and marked with a “0” or a “1” at random. The dates with a “0” were assigned to Optimum® (the control patient management dashboard was turned off) and those with a “1” were assigned to the control patient management dashboard (Optimum® was turned off). Weekdays and weekend/public holiday days were randomized separately.

The principal investigator (TL) was dedicated full-time to this research throughout the recruitment period. He was present in the medical office and had direct access to what was happening to each patient at every moment of the study. He was not involved in management of the study participants. He simply recorded prospectively the time intervals for each patient’s stay in the PED: time of arrival at the PED, evaluation by the triage nurse, the first medical evaluation (by a medical student or a junior physician), the first evaluation by a senior physician, the evaluation by a specialist physician (if applicable), the results of imaging and lab tests (if prescribed), the final medical decision, and discharge.

In addition to the time intervals between the various phases of patient management at the PED, the other variables recorded were age, sex, reason for PED visit, triage level (according to a simplified three-level version of the Pediatric Canadian Triage and Acuity Scale [PaedCTAS] [14]: level 1–2 of the PaedCTAS as level 1 or high-priority level, level 3–4 of the PaedCTAS as level 2 or moderate-priority level, and level 5 of the PaedCTAS as level 3 or low-priority level of the simplified version), and mode of discharge. Five categories of reasons for PED visit were chosen a priori: fever, a respiratory disorder, a digestive tract disorder, trauma, and other reasons.

At the end of the study, the PED staff involved in the study filled out the standardized System Usability Scale (SUS) questionnaire as a guide to the perceived utility of Optimum® and the level of user satisfaction. According to the literature, the SUS score is considered to be very poor if it is less than 51, poor if between 51 and 68, average if 68, good if between 68 and 80.3, and excellent if greater than 80.3 [15–17].

### Statistical analysis

Statistical analyses were performed in Lille University Hospital’s biostatistics unit, using SAS software (version 9.4, SAS Institute Inc., Cary, NC, USA). Firstly, the patients’ characteristics were described. Categorical variables were expressed as the frequency (percentage), and continuous variables were expressed as means and standard deviations (SD) in case of normal distribution or medians with interquartile range [IQR] otherwise. The normality of the data distributions was checked graphically and using the Shapiro-Wilk test. Intergroup comparisons of the total LOS and the various time intervals during patient management times were performed by using an analysis of covariance (ANCOVA) (on log-transformed values; or on rank-transformed values for time interval between the prescription of a consultation with a specialist physician and the consultation itself, and time interval final between evaluation by a senior physician and the end of care) adjusted on priority group at triage (low-priority vs. moderate- and high-priority). Standardized differences and their 95% confidence intervals were calculated as effect sizes; absolute values of 0.2, 0.5 and 0.8 are interpreted as small, moderate and large effect size. Heterogeneity of associations between the various tile intervals and the use of Optimum® according to patient’s priority tirage was tested by adding an interaction term to the model. Curves of mean number of patients present simultaneously in the PED throughout the day were compared between the two groups using a functional analysis of variance and plotted on a graph.

For SUS score, a Spearman’s correlation coefficient was used to evaluate the relation between the age of the PED staff and the SUS score.

## Results

**Fig. 2:**
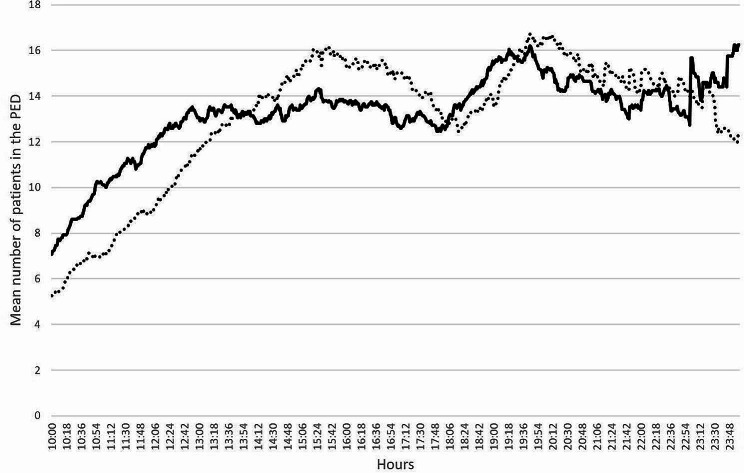
The mean number of patients present simultaneously in the PED, according to the time of day. Full line: the control group; dotted line: the Optimum® group

A 30-day inclusion period enabled us to include 1599 patients (median [IQR] age: 34 months [12–100]; boys: 53%), with 798 in the Optimum® group and 801 in the control group (Table 1). The two groups did not differ significantly with regard to the LOS: 167 min [108–254] in the control group and 172 min [113–255] in the Optimum® group (*p* = 0.46). Likewise, the control and Optimum® groups did not differ significantly with regard to the mean number of patients present simultaneously during the day (*p* = 0.37; Fig. 2).

**Table 1:** Characteristics of the study participants

Patients’ characteristics	Total (*n* = 1599)	Control (*n* = 801)	Optimum® (*n* = 798)
Male sex, n (%)	841/1599 (52.6)	423/801 (52.8)	418/798 (52.4)
Age in months, median [IQR]	34 [12–100]	38 [13–109]	31 [11–93]
Reason for admission:			
Fever, n (%) Respiratory disorder, n (%) Digestive tract disorder, n (%) Trauma, n (%) Other reasons, n (%)	227 (14.2) 260 (16.3) 240 (15.0) 208 (13.0) 664 (41.5)	116 (14.5) 117 (14.6) 131 (16.4) 97 (12.1) 340 (42.4)	111 (13.9) 143 (17.9) 109 (13.7) 111 (13.9) 324 (40.6)
Priority triage			
High priority at triage, n (%)	209 (13.1)	96 (12.0)	113 (14.2)
Moderate priority at triage, n (%)	916 (57.3)	475 (59.3)	441 (55.3)
Low priority at triage, n (%)	474 (29.6)	230 (28.7)	244 (30.6)
Immediate resuscitation, n (%)	14 (0.9%)	8 (1.0)	6 (0.7)
Evaluation by a senior physician			
No, n (%)	538 (33.6)	270 (33.7)	268 (33.6)
One, n (%)	816 (51.0)	418 (52.2)	398 (49.9)
Two, n (%)	245 (15.3)	113 (14.1)	132 (16.5)
Evaluation by a specialist physician from outside the PED			
No, n (%)	1234 (77.2)	624 (77.9)	610 (76.4)
One, n (%)	313 (19.6)	153 (19.1)	160 (20.1)
Two, n (%)	46 (2.9)	19 (2.4)	27 (3.4)
Three, n (%)	6 (0.4)	5 (0.6)	1 (0.1)
Blood test			
No, n (%)	1338 (83.7)	660 (82.4)	678 (85.0)
One, n (%)	249 (15.6)	136 (17.0)	113 (14.2)
Two, n (%)	12 (0.8)	5 (0.6)	7 (0.9)
Imaging examination			
No, n (%)	1160 (72.5)	581 (72.5)	579 (72.6)
One, n (%)	402 (25.1)	205 (25.6)	197 (24.7)
Two, n (%)	33 (2.1)	12 (1.5)	21 (2.6)
Three, n (%)	4 (0.3)	3 (0.4)	1 (0.1)
Treatment			
No, n (%)	1389 (86.9)	699 (87.3)	690 (86.5)
One line, n (%)	201 (12.6)	97 (12.1)	104 (13.0)
Second line, n (%)	9 (0.6)	5 (0.6)	4 (0.5)
Patient outcome			
Discharged to home, n (%)	1238 (77.4)	626 (78.2)	612 (76.7)
Discharged to a short-stay unit, n (%)	175 (10.9)	85 (10.6)	90 (11.3)
Discharged to a hospital ward, n (%)	158 (9.9)	80 (10.0)	78 (9.8)
Left without being seen, n (%)	20 (1.3)	6 (0.7)	14 (1.8)
Discharged to the pediatric intensive care unit, n (%)	8 (0.5)	4 (0.5)	4 (0.5)

Then the two study groups were compared with regard to the various time intervals during patient management (Table 2), and with regard to the priority groups (Supplementary Material 2). For low-priority patients, the time interval between triage and the first medical evaluation was significantly higher in the Optimum® group (*p* = 0.004) than in the control group. For high-priority patients, this median time interval was shorter in the Optimum® group than in the control group (19 [9–41] vs. 23 [11–45] min; *p* = 0.009). For moderate- and high-priority patients, the median time interval between admission and the first evaluation by a senior physician was significantly higher in the Optimum® group (*p* = 0.002), with a smaller difference (75 [49–114] vs. 72 [47–104] min) than for low-priority patients (80 [50–129] vs. 69 [47–94] min), and the median time interval between the final medical decision and discharge was significantly lower in the Optimum® group (10 [0-101] vs. 15 [0-106] min; *p* < 0.03).

**Table 2:** Comparison of time intervals at each stage in the management of children in the PED between the Optimum® group (*n* = 798) and the control group (*n* = 801)

Time interval	Control median [IQR]	Optimum® median [IQR]	Standardized difference (95%CI)
Between admission and evaluation by a triage nurse	15 [8–23]	15 [8–27]	0.12 (0.01 to 0.22)
Between triage and the first medical evaluation*	23 [11–45]	21 [10–50]	0.07 (-0.03 to 0.17)
Between the first medical evaluation* and the first evaluation by a junior physician	29 [19–45]	30 [19–47]	0.07 (-0.15 to 0.30)
Between admission and the first evaluation by a senior physician	71 [47–102]	76 [49–118]	0.22 (0.09 to 0.35)
Between blood sampling and blood test prescription	40 [25–60]	41 [20–75]	0.04 (-0.21 to 0.29)
Between blood sampling and consultation of the blood test results	122 [73–171]	129 [81–180]	0.04 (-0.21 to 0.28)
Between imaging prescription and consultation of results	63 [35–118]	59 [35–100]	-0.06 (-0.25 to 0.13)
Between the prescription of a consultation with a specialist physician and the consultation itself	39 [10–110]	45 [8-106]	0.03 (-0.18 to 0.24)
Between treatment prescription and treatment administration	25 [12–48]	21 [9–43]	0.20 (-0.08 to 0.47)
Between the final evaluation by a senior physician and the end of care	0 [0–97]	0 [0–88]	0.01 (-0.09 to 0.11)
Between the end of care and discharge	33 [16–73]	29 [14–62]	0.13 (0.02 to 0.23)

Medians expressed in minutes. CI: confidence interval. IQR: interquartile range (in minutes)

*First evaluation was sometimes performed by a medical student, rather than a junior physician

Seventy PED staff members (58.3% of those who worked with Optimum® during the study) answered the SUS questionnaire (Table 3). The median [IQR] SUS score was 68 [55–80]. Spearman’s correlation test showed that the SUS score was significantly lower for older PED staff members (-0.46; *p* < 0.001) and significantly higher for staff members who had joined the PED more recently (*p* < 0.001).

**Table 3:** System Usability Scale (SUS) scores

Variables	N	SUS score Median [IQR]
Occupation, n (%) Receptionist, n (%) Nurse assistant, n (%) Nurse, n (%) Medical student, n (%) Junior physician, n (%) Senior physician, n (%)	70 (58) 1 (1.4) 7 (10.0) 8 (11.4) 14 (20.0) 20 (28.6) 20 (28.6)	68 60 [60–60] 55 [48–80] 78.5 [64–81] 75 [65–88] 70 [65–79] 58 [35–65]
Age in years, median [IQR]	28 [25–32]	/
Seniority in months, median [IQR]	24 [6–72]	/
Questionnaire medium used, n (%)		
Internet questionnaire Paper-based questionnaire	65 (92.9) 5 (7.1)	65 [55-77.5] 80 [80–80]

IQR: Interquartile Range

## Discussion

Current results did not show a significant reduction in the median LOS (the primary endpoint) when Optimum® was used. Likewise, the use of Optimum® was not associated with a reduction in the number of children present simultaneously in the PED. However, some significant intergroup differences were observed in specific care duration - especially when considering subgroups of patients with the same triage priority. Interestingly, for high-priority patients, the use of Optimum® was associated with a shorter time to first evaluation by a senior physician and an earlier final decision on the discharge destination. The longer time interval between admission and first assessment by a senior physician for patients in the Optimum® group, although less for high-priority than for low-priority patients, may be explained by the few minutes of latency Optimum® has in retrieving data from the standard management dashboard.

One potential interpretation of no LOS change overall is that the initial triage was about right and that the PED’s processes were relatively good to begin with. Although triage is a key strategy for managing patient flow in the PED [18, 19], staff sometimes make arbitrary decisions and do not comply with the triage decisions. To the best of our knowledge, the present study is the first to have evaluated a software tool for secondary prioritization in the ED. Many studies have reported the value of triage on ED entry [4, 18, 19]. But secondary prioritization after the first medical evaluation might also help to reduce the patients’ LOS. By prioritizing the patients, Optimum® made it easier to track triage groups. As expected, low-priority patients waited longer to be seen by a physician. We noticed that strict compliance with Optimum®^’^s recommendations resulted in a wait of several hours for low-priority patients when higher-priority patients continued to arrive in the PED. The Optimum® algorithm prioritized low-priority patients only when their LOS was above the 95th percentile for patients admitted for the same reasons. Not re-evaluating a patient who had been waiting for many hours was sometimes psychologically tough for both the individuals concerned and the PED staff. Low-priority patients can feel vulnerable when staff pay more attention to other patients [20]. The time for low-priority patients to be prioritize should be optimized in the future [21]. Another way of managing this problem would be the creation of a fast-track team for the treatment of trauma or for handling non-urgent ED attendance- a well-known strategy for shortening the LOS [18, 22, 23]. We hypothesize that once a predetermined threshold for the patients in the PED would be reached, Optimum® could suggest the activation of a dedicated fast-track team for prioritized patients. Better management of non-urgent ED attendance visits might also reduce the number of patients who leave the ED without being seen, which is directly linked to the LOS [24, 25].

We also noted that use of Optimum® was associated with a longer time interval between admission and evaluation by a senior physician. It is well known that the presence of trainee physicians in teaching hospitals is linked to longer LOS [26, 27]. In the present study, medical students and junior physicians followed the algorithm’s recommendation of seeing new patients rather than referring previously seen patients to a senior physician (except high priority patients). This aspect of the algorithm needs to be refined. In many cases, additional tests and/or treatments are prescribed only after the patient has been evaluated by a senior physician; the LOS could therefore be shortened by reducing the time interval between admission and evaluation by a senior physician.

The SUS score appeared to depend on age and seniority. One can argue that older staff find it more difficult to adapt to new technologies and new work habits. Furthermore, more experienced staff tend to criticize the need for prioritization more readily. The present study highlighted some key points for improvement of the software’s interface: entering the staff member’s initials next to the name of the patient being cared for, the patient’s location in the PED, and the implementation of a fast track. At each reassessment an adjustment of the patient’s state of health, which can vary over time in a dynamic environment such as the PED, could be added. Lastly, we noted that the response rate was lower among nursing and auxiliary staff than among physicians; in the future, we intend to ask all staff members about their views and their suggestions for improving Optimum®.

### Strengths and limitations

The strength of this study lies in the design of the study and a precise determination of each time interval. As the days were randomized and not the patients, the randomized software was allocated by day, thus avoiding any randomization deviations. The presence of a dedicated full-time researcher enabled data to be collected accurately, thus avoiding any measurement bias for time intervals. This approach generated reliable data on actual time intervals. Retrospective data collection, which could have made possible the inclusion of a much larger number of patients, would not have made it possible to estimate these time intervals with as much precision, due to *a posteriori* data entry or *a posteriori* patient sticker movement. Moreover, there were no missing data in our dataset.

This study had some limitations. Firstly, the principal investigator was not blinded to the software used (i.e., Optimum® or standard dashboard). However, this did not influence patient flow, since he was only a data collector and was not involved in the management of study participants. Secondly, the end of study coincided with a national lockdown period during the epidemic of coronavirus disease 2019 in France; the number of PED visits was below average during this time. In contrast, the start of study coincided with an unexpectedly late epidemic of bronchiolitis, which probably lead to a high number of PED visits. Thirdly, our target of a 15-minute reduction LOS was probably too ambitious, when compared with most of the studies of throughput in the literature [4, 18]. This point, combined with a smaller than expected number of patients, could explain the absence of any significant difference observed. Fourthly, staff allocation bias is unlikely because the number of nurses and physicians was small and the study long enough to allow random allocation of staff between days using the standard software and days using the Optimum® software. Fifthly, staff take-up of the software may have been uneven or insufficient. Evaluation using a learning curve would have been useful but was not carried out. Sixthly, the SUS score may have been lower if we consider that the staff most interested in Optimum completed the questionnaire. Lastly, during the study period, triage was performed by nurses who also had to collect blood samples or administered drugs; none of the nursing staff members was dedicated to triage in the PED. The time intervals between admission, triage and imaging/lab tests could perhaps be reduced by having a dedicating triage nurse, although that was not possible at the time of the study.

## Conclusions

The Optimum® group and the control group did not differ with regard to the overall LOS in the PED. Interestingly, however, the use of Optimum® appeared to influence certain phases in patient care - especially for high-priority patients. Refinement of the Optimum® software (e.g. through machine learning) might make it useful in the ED, as has already been demonstrated for triage with other software tools [28].

### Electronic supplementary material

Below is the link to the electronic supplementary material.

Supplementary Material 1

## Data Availability

No datasets were generated or analysed during the current study.
