# Supplementary figures and images for: Randomized controlled open-label trial to evaluate prioritization software for the secondary triage of patients in the pediatric emergency department
Source: Int J Emerg Med. 2024 Apr 8;17:53. doi: 10.1186/s12245-024-00623-3 (PMC11000356; doi:10.1186/s12245-024-00623-3)

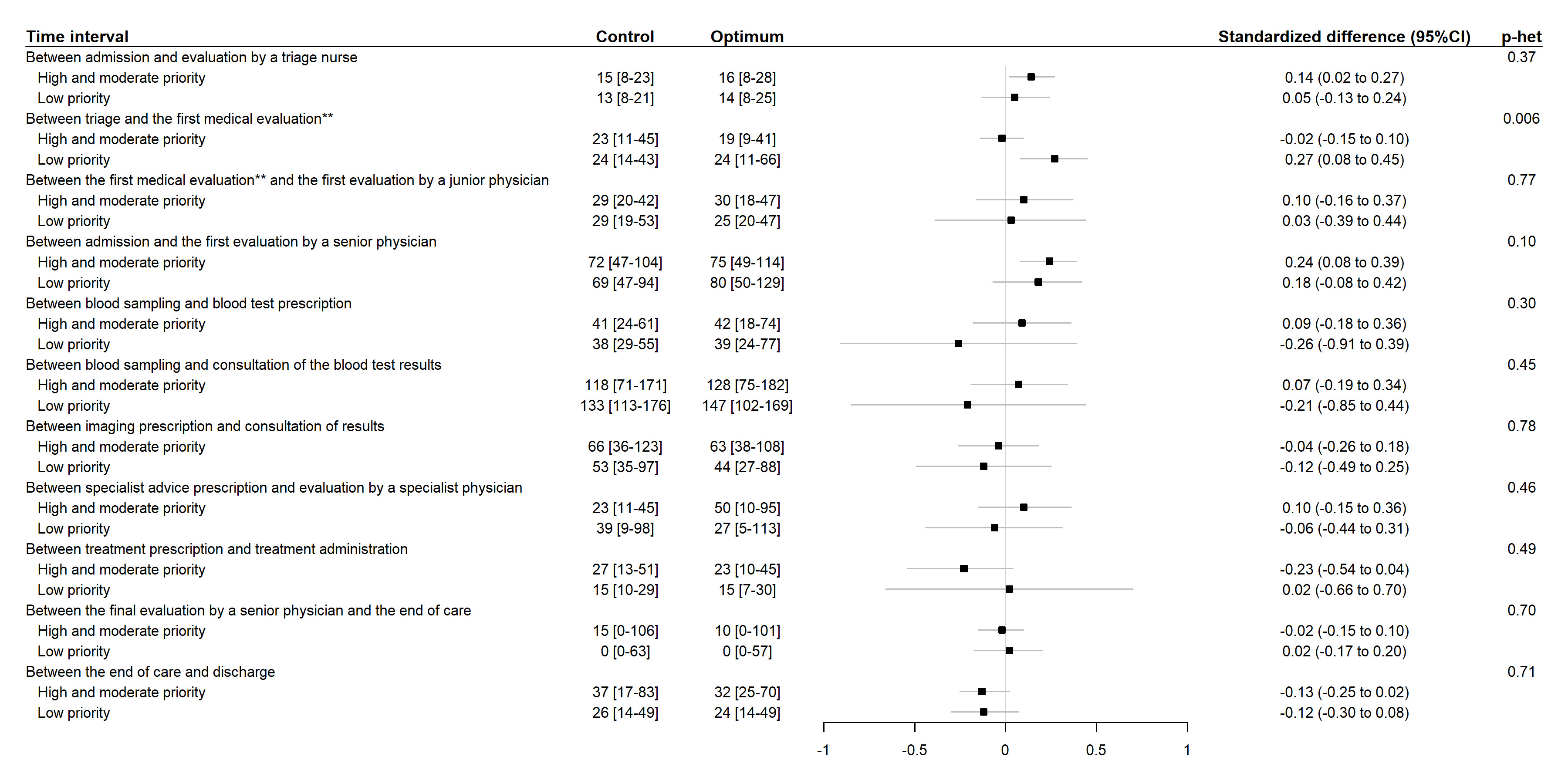

Supplement: Supplementary file 1 — Supplementary Material 1 [file 12245_2024_623_MOESM1_ESM.jpg]
